# Age-corrected measurement of extracellular volume fraction in remote normal myocardium is correlated with extent of risk area in AMI patients

**DOI:** 10.1186/1532-429X-16-S1-O20

**Published:** 2014-01-16

**Authors:** Yoshitaka Goto, Masaki Ishida, Tatsuro Ito, Mio Uno, Motonori Nagata, Kakuya Kitagawa, Shiro Nakamori, Kaoru Dohi, Masaaki Ito, Hajime Sakuma

**Affiliations:** 1 Radiology, Mie University Hospital, Tsu, Mie, Japan; 2 Cardiology, Mie University Hospital, Tsu, Mie, Japan

## Background

Reduction of post-contrast T1 in remote normal myocardium was recently reported in patients with AMI, indicating that extracellular matrix expansion occurs in remote myocardium early after MI. Such extracellular matrix expansion observed in non-infarcted myocardium without edema seems to be one of key factors causing LV remodeling after MI. However, since the previous study only determined post-contrast T1, alteration of extracellular volume fraction (ECV) in remote myocardium early after MI is not investigated quantitatively. Consequently, we sought to quantify ECV in remote normal myocardium in AMI patients using T1 mapping before and after gadolinium contrast with hematocrit and heart-rate corrections, and investigate the association between ECV expansion in remote normal myocardium and extents of infarction and area at risk.

## Methods

Sixteen reperfused AMI patients (13 men, 65 ± 13 years, 7.0 ± 2.5 days after onset), age- and sex-matched 16 OMI patients (4.1 ± 5.7years after onset) and age- and sex-matched 16 control subjects with normal CMR findings who underwent CMR including cine, T2WI (for AMI patients only), LGE and T1 mapping were retrospectively studied. T1 mapping was performed using a modified Look-Locker inversion recovery sequence. ECV was determined according to myocardial and blood T1 values measured before and after administration of gadolinium contrast and hematocrit measures. T1 values were corrected for heart rate. Risk size and infarct size was determined on T2WI and LGE images, respectively, in AMI patients. Age-related changes in T1 and ECV were also corrected using a least square fitted line obtained in control subjects.

## Results

**Figure 1:**
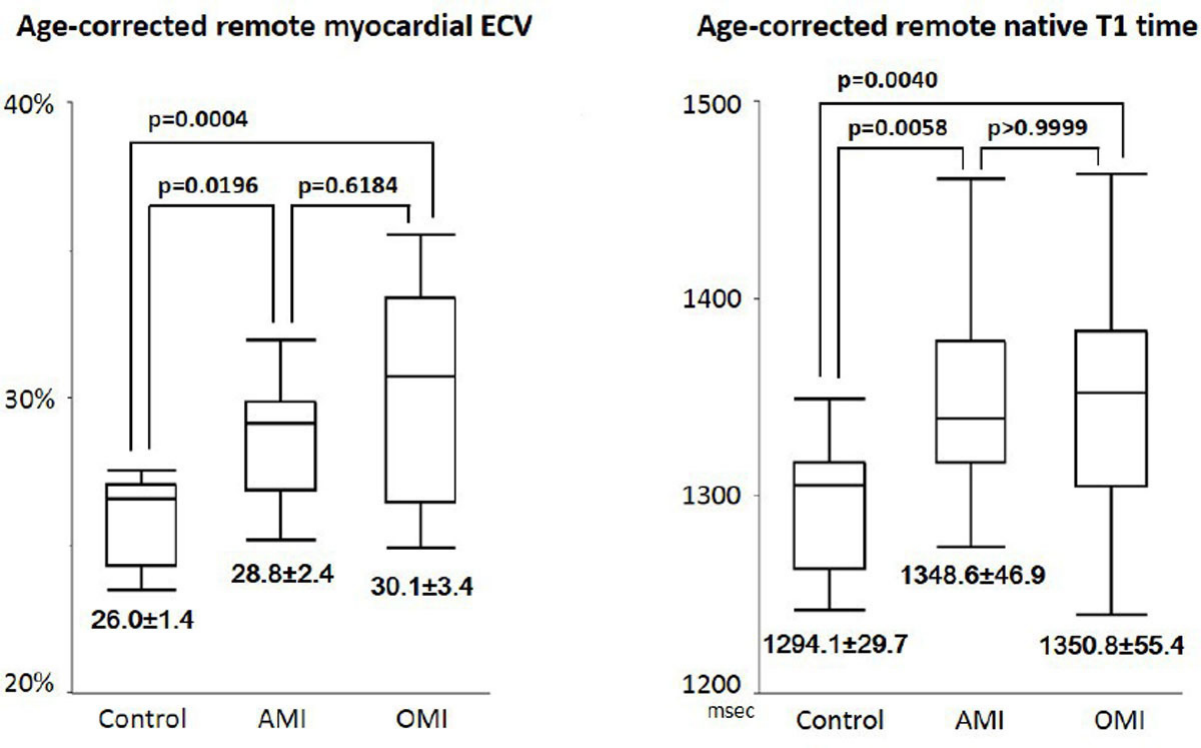


**Figure 2:**
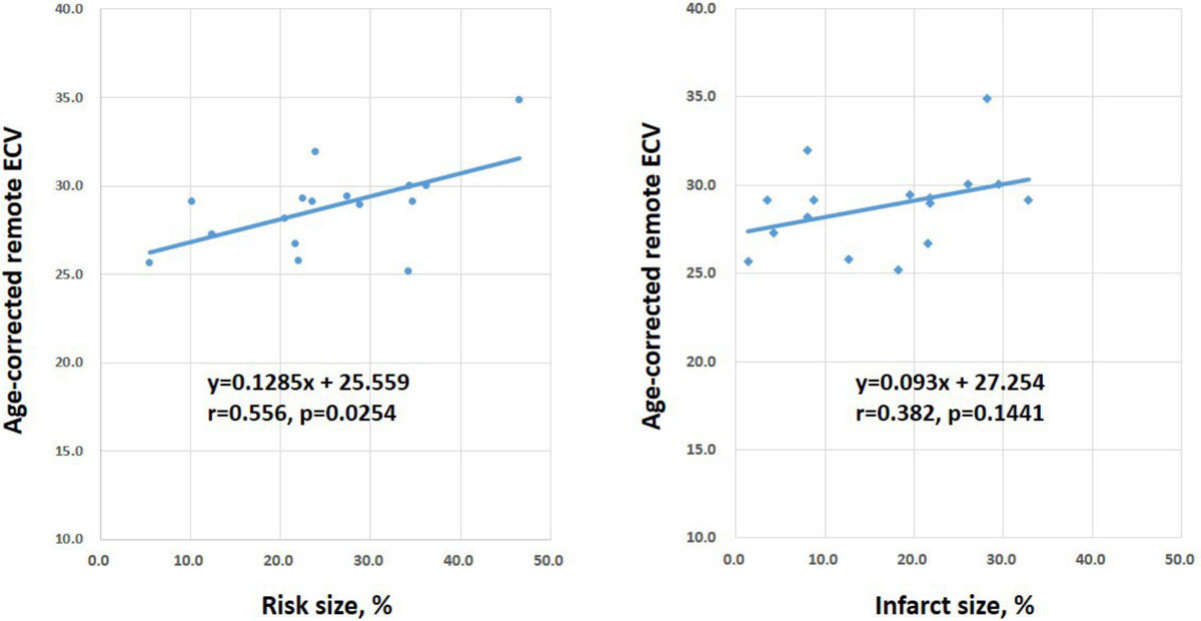


Both ECV and native T1 were strongly associated with age in control subjects (y = 0.12x+18.16, r = 0.689, p = 0.003 and y = 1.5175x+1202.6, r = 0.503, p = 0.047, respectively). Age-corrected remote myocardial ECV was significantly larger in AMI patients (28.8 ± 2.4%) than in normal subjects (26.0 ± 1.4%, p = 0.0196), whereas age-corrected native remote myocardial T1 was significantly longer in AMI patients (1349 ± 47 ms) than in control subjects (1294 ± 30 ms, p = 0.0058). However, no significant difference was observed between AMI and OMI patients both in ECV and native T1 in remote myocardium (28.8 ± 2.4% vs. 30.1 ± 3.4%, p = 0.6184 and 1349 ± 47 ms vs. 1350 ± 55 ms, p > 0.9, respectively) (Figure 1). Age-corrected remote myocardial ECV in AMI patients was moderately correlated with risk size (r = 0.56, p = 0.0254) but only weakly with infarct size (r = 0.38, p = 0.1441) in AMI patients (Figure 2).

## Conclusions

ECV expansion up to 30% in remote normal myocardium occurs early after AMI onset and persists in chronic state. ECV expansion in remote normal myocardium is correlated with the extent of risk area in AMI patients.

## Funding

I have no conflict of interest.

